# Effects of Parental Migration on Life Satisfaction and Academic Achievement of Left-Behind Children in Rural China—A Case Study in Hubei Province

**DOI:** 10.3390/children5070087

**Published:** 2018-06-27

**Authors:** Shujuan Song, Chunfeng Chen, Aiguo Zhang

**Affiliations:** 1 College of Education, Huanggang Normal University, Huanggang 438000, China; 2 Guankou Middle School, Huanggang 438000, China; chenchunfeng2233@163.com (C.C.); zhangaiguo5678@163.com (A.Z.)

**Keywords:** parental migration, left-behind children, life satisfaction, academic achievement, rural China

## Abstract

In the rural areas of China, there is a high occurrence of parental migration, wherein adults are flushed into urban areas to search for employment opportunities, leading to millions of left-behind children (LBC) in rural China. LBC attracts more attention from the social community and Chinese government. Here, we compared the life satisfaction and academic achievement of left-behind children (LBC) and non-left-behind children (NLBC) in rural regions that send out migrant labor in Hubei province, central China. We investigated 1031 LBC and 992 NLBC students in grades 4 to 9 in ten elementary and four middle schools, using a structured questionnaire including sociodemographic characteristics, life satisfaction, and academic achievement scores. The results showed that LBC have a lower life satisfaction and lower academic achievement than NLBC (*p* < 0.01). Meanwhile, as the child’s age at separation from parents decreased, their life satisfaction decreased. Additionally, correlations were observed between life satisfaction and academic achievement scores in LBC (*p* = 0.004) as well as in NLBC (*p* = 0.064). Collectively, these findings provide novel insights into a comprehensive understanding of LBC and suggest that the life satisfaction levels of LBC should be improved in rural China.

## 1. Introduction

With rapid economic and social development, more and more residents from rural areas have been moving into urban areas in order to seek better jobs and human development opportunities [1]. These labor population movements have led to 61 million left-behind children (LBC) in rural China, an issue which is attracting more and more attention from the social community and Chinese government [2]. A recent review by Zhao et al. [3] estimated that 29 million LBC have experienced separation from their parents. Although several efforts from community supports have been made to resolve these problems induced by rural migration, it is still a large challenge for school managers to improve the development of LBC in rural China [4,5].

During the past few decades, researchers have suggested that these children’s development is influenced by parental migration [6,7,8]. A great deal of literature has demonstrated that parental migration has a negative or positive influence on the psychological development of children, especially in developing countries like China, the Philippines, and Mexico [6,7]. For example, Zhang et al. [8] compared the different total energy intake between children left behind and children from intact families in rural China and suggested that LBC consumed lower-protein and higher-fat food compared with non-left-behind children (NLBC). Meanwhile, Zhao et al. [7] found that LBC suffered from higher levels of anxiety and poorer living conditions compared to NLBC. Li et al. [9] determined the psychosocial health and living quality of LBC in a remote city in southwest China named Guiyang, and the results revealed that parental migration reduced the physical and mental health of children. A recent study conducted by Hu et al. [10], who compared the risks of behavioral problems among migrant children, local children, and LBC in China, and found that parental migration contributed to higher behavior problems of children.

Life satisfaction is one of the features of well-being, which plays an important role in shaping a child’s psychological development and in evaluating the quality of a child’s life [11]. Previous studies have demonstrated that life satisfaction is related to several advantageous outcomes, such as social relationships, job opportunity, and occupational success [6,7,12]. Barger et al. [13] determined the relative contributions of socioeconomic status, health, and social relationships to life satisfaction in the United States. Another evidence indicated that life satisfaction was related to educational performance. For instance, Antaramian [14] evaluated college student performance and found that students with higher life satisfaction had the highest grade point averages. However, limited studies have focused on determining the effect of parental migration on the life satisfaction of LBC students at elementary school and middle school levels.

Based on the comprehensive investigation of the relationship between life satisfaction and the academic achievement of LBC, school management will be improved significantly [12,13,14]. The psychological life satisfaction and academic achievement of children are vital for the development of LBC, which is an important issue because children represent the future of our country [15,16]. To date, the number of LBC in rural regions of China has increased dramatically [17]. Vanluot and Badat [18] studied psychological life satisfaction among LBC of labor migrant parents in rural northern Vietnam and revealed that having migrant parents had negative influences on the life satisfaction of LBC. Moreover, Li et al. [19] surveyed junior high school students and explored the effect of parental migration on the academic achievement of left-behind middle school students in rural China, demonstrating that parental migration had a dramatic negative impact on the academic achievement of junior high school students. Unfortunately, the relationship between life satisfaction and the academic achievement of LBC student is not well understood. Moreover, no study has focused on examining whether LBC students suffering from a lower life satisfaction level report lower academic achievement than NLBC students.

To narrow this gap in knowledge, the general objective of the present study was to explore the effect of parental migration on the psychological life satisfaction and academic achievement of LBC in rural China. To address this topic, the specific objectives were to: (1) determine the psychological life satisfaction and academic achievement of LBC (e.g., including gender and grade) shaped by parental migration; (2) investigate the relationship between life satisfaction and the academic achievement scores of LBC in rural China. The results from this study will contribute to a comprehensive understanding the life satisfaction and educational status of LBC influenced by parental migration in rural areas of China.

## 2. Materials and Methods

### 2.1. Participants

The study area was located in Hubei province in central China. Hubei province is one of the largest cross-province emigrant population province and has one of the largest numbers of LBC in China [1]. In this study, a survey was conducted between May and September 2016. The participants consisted of 2023 students (LBC = 1031, NLBC = 992) from 10 elementary and four middle schools. All participants were in grades 4 to 9, aged between 8 and 16 years. The participant response rate was 89.5% (2023/2260). Questionnaires that had 10% (or more) missing items were eliminated from the study. To determine the effects of different grades, grades 4–9 were selected. Grades 4–6 and 7–9 were listed as elementary school and middle school levels, respectively. This study was approved by the Ethics Committee of Huanggang Normal University (Project 12/2016).

### 2.2. Procedure

Approval for this study was obtained from the ethical review board at the schools. All LBC and NLBC students volunteered for this study and provided survey information revealing their agreement to participate. To ensure that all the participants were capable of comprehensive understanding and finishing the questionnaires, a pilot test among students was conducted in grade 4.

### 2.3. Measures

Two dependent variables (e.g., life satisfaction score and academic achievement score) were determined in this survey. Briefly, life satisfaction was most frequently used to explore a youth’s perceived quality of life [20]. Subsequently, life satisfaction was measured using the satisfaction with life scale (SWLS) developed by Pavot and Diener with five items [21]. Response categories were as follows: strongly agree (score = 7), agree (score = 6), slightly agree (score = 5), neither agree nor disagree (score = 4), slightly disagree (score = 3), disagree (score = 2), and strongly disagree (score = 1). The life satisfaction score for each LBC and NLBC student was calculated based on the seven levels, and the total scores ranged from 5 to 35. The Chinese version of the SWLS, which demonstrated good reliability and validity, was used with Cronbach’s alpha 0.78 [22]. In the present study, academic achievement was measured by standardized Chinese, Mathematics, and English test scores provided by 42 teachers. For elementarily school, the total score for each course was 100, while it was 120 for middle school. Therefore, the total academic achievement scores of each elementarily school student and middle school student were 300 and 360, respectively.

### 2.4. Statistical Analysis

Data were expressed as means and standard deviation (e.g., means ± SD). Descriptive statistics were used to assess the distribution of samples in this study. The difference of life satisfaction and academic achievement parameters between LBC and NLBC was compared by using one way-ANOVA followed by Tukey’s HSD (honest significant difference) test (SPSS version 20.0, SPSS Inc., Chicago, IL, USA). To explore the relationship between life satisfaction and academic achievement, Pearson’s correlation analysis was also performed between life satisfaction and academic achievement scores by the IBM SPSS statistics 20.0 (SPSS Inc., Chicago, IL, USA).

## 3. Results

### 3.1. Sample Distribution

In the present study, the sample description and distribution are summarized in Table 1. For participants, the sampling numbers was 2023, the proportion of LBC was 50.96% (1031/2023). As shown in Table 1, 50.23% of all participants were boys, while 49.77% were girls. Meanwhile, elementary school students composed 41.42% of the sample. A significant difference in grade 7 was found between the LBC and NLBC (χ^2^ = 8.124, *p* = 0.004). However, chi-square tests showed that there were no significant differences between the two groups in terms of gender (*p* > 0.05) and educational level (*p* > 0.05).

### 3.2. Life Satisfaction between LBC and NLBC

Table 2 shows the results of the comparison of life satisfaction between LBC and NLBC. In total, a significant difference in life satisfaction was observed between the LBC and NLBC (*p* = 0.013). Marginal significant gender differences between LBC and NLBC could be observed (*p* = 0.145, *p* = 0.056). Clearly, for grades 7 and 8, a significant difference in life satisfaction was observed between the LBC and NLBC (*t* = 2.277, *p* = 0.023; *t* = 2.385, *p* = 0.018). Furthermore, LBC in middle school also had a significantly lower life satisfaction score (*t* = 2.380, *p* = 0.017) (Table 2).

### 3.3. Academic Achievement between LBC and NLBC

Table 3 shows the academic achievement (e.g., gender, grade, and educational level) between LBC and NLBC. Notably, LBC had a significant lower academic achievement score than that of NLBC (*t* = 2.392, *p* = 0.017). There was no significant difference of academic achievement between the two groups in terms of grade and educational level (*p* > 0.05 in both cases). For gender, being left behind had significant effects on the academic achievement of girls (*t* = 2.224, *p* = 0.026).

Table 4 shows the comparison of the life satisfaction and academic achievement of LBC with three separation age levels (e.g., aged < 3, 3 < aged < 6, and aged > 6). As the child’s age at separation from parents decreased, their life satisfaction decreased. The life satisfaction score was significantly lower for children whose parents were away before they reached their third birthday (M = 20.30; SD = 6.644), followed by children whose parents left when they were aged 3 to 6 years (M = 21.10; SD = 6.141) (*F* = 3.900, *p* < 0.05). For academic achievement, children whose parents were away when they were aged more than 6 years reported significantly higher academic achievement scores (M = 211.19; SD = 61.221) (*F* = 3.891, *p* < 0.05) (Table 4). Moreover, a significant difference was also observed between children whose parents left when they were less than 3 years old (M = 205.76; SD = 60.985) and 3 to 6 years old (M = 197.02; SD = 59.898). Further analyses suggested that separation age has a consistent and significant effect on the life satisfaction and academic achievement of left-behind children (LBC) in rural China.

### 3.4. The Relationship between Life Satisfaction and Academic Achievement

**Figure 1:**
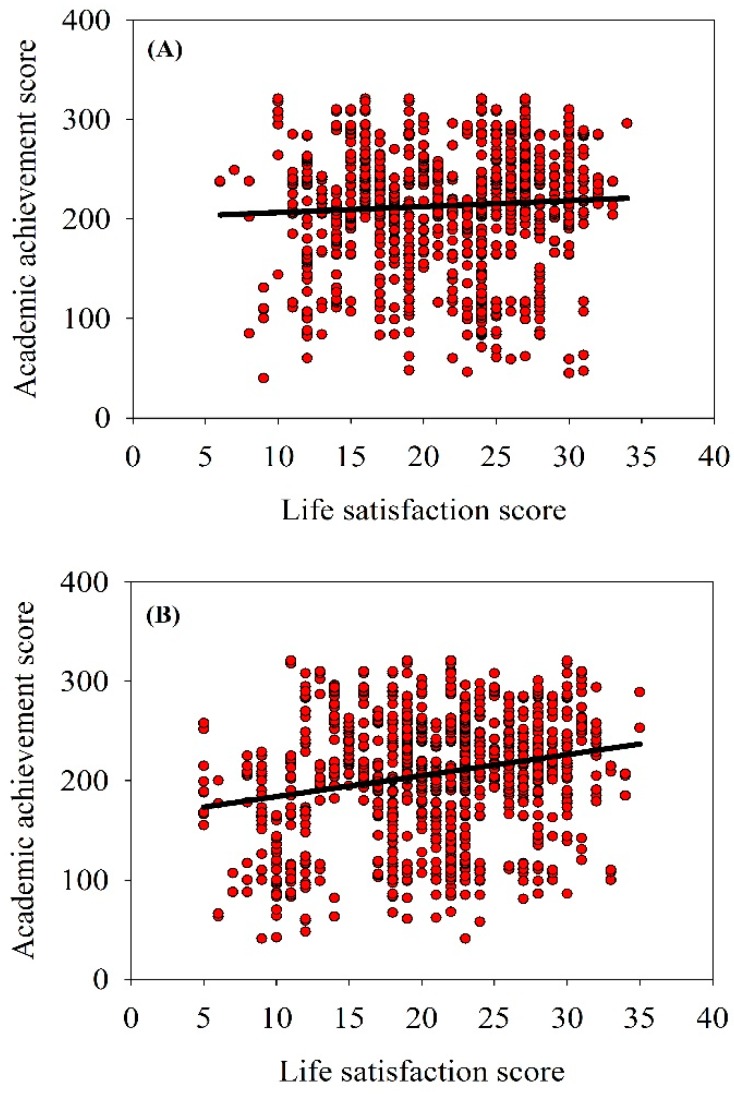
The relationship between life satisfaction and academic achievement of (**A**) non-left-behind children and (**B**) left-behind children (LBC) in rural China (*n* = 2031).

As shown in Figure 1, life satisfaction was significantly positively correlated with academic achievement scores of LBC (y = 2.112, X + 162.83, *r* = 0.091, *p* = 0.004, *n* = 1031), however, a weak relationship was found between life satisfaction and academic achievement scores of NLBC (y = 0.5982 X + 200.04, *r* = 0.059, *p* = 0.064, *n* = 992) (Figure 1).

## 4. Discussion

Labor migration flows have impacts on family structures, which lead to the separation of family members and their children [1,5,8]. To date, LBC have become increasingly prominent in developing countries like China, and they represent a significant challenge for family and society [9,10]. Here, the present study investigated the academic achievement and life satisfaction of LBC students in rural China. Results indicated that LBC had lower life satisfaction and academic achievement than NLBC. Meanwhile, LBC students with higher life satisfaction scores had a high academic outcome. The following issues should be discussed.

During the past few decades, with the rapid urbanization of China, a large number of residents living in China’s rural towns and villages have migrated to developed countries or wealthier regions to seek good job opportunities and higher income [1]. As a consequence, the soaring number of left-behind children (also called Liu-Shou-Er-Tong) has raised dramatically [1,3]. Previous studies have demonstrated that LBC suffer from physiological and psychological health problems such as high levels of depression, anxiety, suicide, innutrition, smoking, and bullying behaviors [7,23]. Parental migration is a vital factor affecting children’s development and behavior due to a lack of caring and monitoring [23]. In recent years, LBC have been recognized as a significant social problems, and they have received increasing attention from China and all over the world [23]. Although many studies have highlighted that LBC represent an important challenge in our society, the impacts of parental migration on the life satisfaction and academic achievement of LBC in rural China still remain unclear. To address this issue, we compared the life satisfaction and academic achievement of LBC and NLBC in Hubei province, central China.

Life satisfaction plays a crucial role in the development of children’s mental health [24]. The life satisfaction of children is dramatically influenced by good family communication, particularly concerning the parents’ connectedness, because parents can help their children to adjust to hardship and face stressful conditions during their daily life [25]. During the past few decades, numerous studies have investigated life satisfaction in children from Korean [26], Qatar [27], Iran [28], Germany [29], Mexico [6], North America [25], and China [16]. Notably, family and school are two important settings for a child’s physiological and psychological development [24,29]. The findings of this study showed that LBC have lower life satisfaction than NLBC, which could be due to the absence of parental care. This is consistent with the report by Cortina [30], who investigated the impact of international migration on children’s life satisfaction in the cities of Tirana, Albania, and Quito, Ecuador, and suggested that parental migration had a negative influence on LBC’s life satisfaction. This was because LBC separated from their parents faced several challenges such as learning difficulty and emotional disturbance, while they could not communicate with their parents quickly and effectively. Mostly, LBC cannot adjust their mind correctly without parental guidance. Moreover, LBC in rural China experience higher levels of emotional anxiety and poorer living conditions [7]. In addition, it was found that the earlier the separation from their parents, the lower the life satisfaction of LBC. In our study, the trend towards increasing life satisfaction with decreasing grade was consistent with a previous study documenting the relationship between satisfaction and age, which found that life satisfaction decreases with increasing age [25]. 

Interestingly, the present results also suggested that paternal absence has a greater influence on left-behind girls as compared to boys. This is because girls are more sensitive to interpersonal relationships and are expected to take on more heavy housework [31]. Due to the rural culture of China, left-behind girls need to take care of their siblings when their parents go out to work. Another reason is that girls normally receive more less care and support from family and relatives due to the boy bias [31]. This result is consistent with the universal phenomenon of rural China in that family members usually love boys than girls [32]. In rural China, the girls are expected to take care of their grandparents and complete daily housework after school [33]. In many villages, female students drop out of school because their families cannot afford the school expenses for both girls and boys [34]. Girls may suffer more mental challenges during parental migration, and they hope for more parent-child interaction, especially when they are young [35]. Therefore, sex-specific abortions remain extremely in villages, and boys are regarded as superior to girls, which may enhance the negative effects of paternal absence on left-behind girls. Taken together, the results of the LBC life satisfaction scores indicated that parental migration can reduce the life satisfaction of LBC.

Another potential consequence of parental migration is the influence on children’s educational aspirations [36]. In the present study, the results revealed that parental migration has negative impacts on the academic achievement (based on standardized Chinese, Mathematics, and English test scores) of LBC. This is not consistent with the recent survey conducted by Bai et al. [37], who found that parental migration has significantly positive impacts on the academic achievement of LBC, especially for poorer performing LBC. Because the household income will increase when the parents leave home for better jobs, more learning resources and facilities can be provided to LBC by the higher household income. On the contrary, Lu [38] indicated that LBC are worse off in educational attainment than NLBC in Mexico and Indonesia. The present results are consistent with those of Li et al. [19], who found that parental migration has a significant negative influence on the academic achievement of junior high school students. Parent-school communication is essential for student education [39]. The lower academic achievement of LBC may be related to other factors, such as poor psychosocial health [2] and malnutrition [40], poor living quality [19], and school education quality.

Life satisfaction is a vital determinant of academic success [41]. However, the literature exploring the relationship between students’ academic achievement and life satisfaction has shown conflicting results, while the main reasons behind these results have not been evaluated. Previous works have suggested that parental migration has a negative impact on the education and life satisfaction of the children left behind [36,38]. Unfortunately, research that addresses the relationship between the life satisfaction and academic achievement of LBC students is limited. This is the first study to be conducted in rural China to address the relationship between the life satisfaction and academic achievement among LBC students. More notably, this research found that LBC students with higher levels of psychological life satisfaction also showed higher levels of academic achievement. This result is consistent with prior research conducted by Antaramian and Lee [41], who revealed that life satisfaction was significantly correlated with most of the academic outcomes. Therefore, a higher life satisfaction may be help students to reduce academic stress and increase academic self-efficacy. Crede et al. [42] demonstrated that academic achievement is positively correlated with the life satisfaction of high school students, and an association between life satisfaction and academic achievement was observed in students whose mothers had a higher education diploma than their own children. Lv et al. [39] found that parents influence the attitude of elementary school children toward academic achievement as well as parent–school communication. Additionally, life satisfaction has less influence on the academic achievement of NLBC.

However, the relationship between life satisfaction and academic achievement was evaluated by a meta-analysis of 47 individual works with a total of 38,946 participants, and the results suggested that low-achieving students do not necessarily report low life satisfaction [43]. Recently, Dimitrova et al. [44] suggested that the life satisfaction and school achievement of young students were related to their ethnic socialization and ethnic identity. From a global point of view, the relationship of psychological life satisfaction and school achievement may be structured by socialization agents [45]. Taken together, the findings of this research highlighted that parental migration has a negative impact on both the life satisfaction and academic achievement of LBC in rural China. The results also suggested that LBC life satisfaction should occupy an important position in schools. Although this study helped us to understand the effects of parental migration on LBC in China, LBC with different levels of life satisfaction should evaluated in the future. Moreover, further studies are encouraged to generate a model for parental migration, LBC life satisfaction, and LBC academic achievement using longitudinal studies [46]. 

## 5. Conclusions

In summary, the present study systematically investigated the effects of parental migration on the life satisfaction and academic achievement of left-behind children (LBC) in rural Hubei province, China. The results showed that LBC had lower life satisfaction and academic achievement than NLBC. Paternal absence has a greater influence on left-behind girls than boys. Meanwhile, the younger the age at separation, the lower the life satisfaction of a child would manifest. Furthermore, a significant correlation was found between the life satisfaction and academic achievement scores in LBC and in NLBC. Taken together, these results from the present study will contribute new insights toward a comprehensive understanding of the life satisfaction levels and academic achievement of LBC in rural China.

## Figures and Tables

**Table 1:** Description of the participant characteristics in this study.

Number (*n*) and Frequency (%)								
Variable	Overall		LBC		NLBC		χ^2^	*p*
	*n* = 2023	100%	*n* = 1031	51%	*n* = 992	49%	0.752	0.386
**Gender**								
Male	*n* = 1000	100%	*n* = 509	50.9%	*n* = 491	49.1%	0.580	0.446
Female	*n* = 991	100%	*n* = 502	50.7%	*n* = 489	49.3%	0.227	0.634
**Grade**								
Grade 4	*n* = 257	100%	*n* = 124	48.2%	*n* = 133	51.8%	0.315	0.575
Grade 5	*n* = 252	100%	*n* = 139	55.2%	*n* = 113	44.8%	2.683	0.101
Grade 6	*n* = 329	100%	*n* = 160	48.6%	*n* = 169	51.4%	0.246	0.620
Grade 7	*n* = 386	100%	*n* = 207	56.3%	*n* = 161	43.8%	8.124	0.004
Grade 8	*n* = 403	100%	*n* = 199	50.3%	*n* = 197	49.7%	0.089	0.766
Grade 9	*n* = 396	100%	*n* = 182	46.8%	*n* = 207	53.2%	0.836	0.175
**Educational level**								
Elementary school (grades 4–6)	*n* = 838	100%	*n* = 423	50.5%	*n* = 415	49.5%	0.076	0.782
Middle school (grades 7–9)	*n* = 1185	100%	*n* = 588	51.0%	*n* = 565	49.0%	1.030	0.310

Note. LBC and NLBC represent left-behind children and non-left-behind children, respectively. The number of gender is less than the total participants’ number because some children did not report their gender data. *p* < 0.05 is considered significant.

**Table 2:** Comparison of life satisfaction of left-behind children (LBC) and non-left behind children (NLBC) in rural China.

Variable	LBC (M, SD) (*n =* 1031)	NLBC (M, SD) (*n =* 992)	*t*	*p*
**Total**	20.98, 6.273	21.66, 5.877	2.498	0.013
**Gender**				
Male	20.99, 6.313	21.56, 5.975	1.460	0.145
Female	21.05, 6.264	21.79, 5.772	1.910	0.056
**Grade**				
Grade 4	22.14, 5.623	22.75, 5.245	0.907	0.365
Grade 5	22.74, 7.324	22.50, 6.235	-0.272	0.786
Grade 6	20.64, 6.467	21.64, 6.504	1.400	0.162
Grade 7	20.59, 5.993	21.51, 5.332	2.277	0.023
Grade 8	20.43, 5.450	21.71, 5.246	2.385	0.018
Grade 9	20.07, 5.990	20.67, 6.317	0.968	0.334
**Educational level**				
Elementary school (grades 4–6)	21.77, 6.584	22.23, 6.058	1.059	0.290
Middle school (grades 7–9)	20.43, 5.992	21.24, 5.712	2.380	0.017

Note. Data are expressed as means (M) and standard deviations (SD). LBC and NLBC represent left-behind children and non-left-behind children, respectively. *p* < 0.05 was considered significant.

**Table 3:** Comparison of academic achievement of left-behind children (LBC) and non-left behind children (NLBC) in rural China.

Variable	LBC (M, SD) (*n =* 1031)	NLBC (M, SD) (*n =* 992)	*t*	*p*
**Total**	206.39, 60.925	212.79, 59.612	2.392	0.017
**Gender**				
Male	207.71, 60.385	212.49, 58.254	1.276	0.202
Female	205.00, 60.954	213.61, 61.073	2.224	0.026
**Grade**				
Grade 4	197.86, 58.272	203.32, 57.335	0.756	0.450
Grade 5	196.94, 58.030	206.21, 62.662	1.217	0.225
Grade 6	198.88, 64.010	204.72, 63.429	0.830	0.407
Grade 7	211.96, 62.405	219.30, 57.932	1.178	0.240
Grade 8	208.09, 60.141	216.39, 56.108	1.438	0.151
Grade 9	217.13, 59.288	220.23, 59.463	0.518	0.605
**Educational level**				
Elementary school (grades 4–6)	197.94, 60.592	204.68, 60.294	1.604	0.109
Middle school (grades 7–9)	212.22, 60.724	218.63, 57.803	1.863	0.063

Note. Data are expressed as means (M) and standard deviations (SD). LBC and NLBC represent left-behind children and non-left-behind children, respectively. *p* < 0.05 was considered significant.

**Table 4:** Comparison of the life satisfaction and academic achievement of left-behind children (LBC) in rural China.

Variable		Life Satisfaction (M, SD)	Academic Achievement (M, SD)
Separation age	Aged < 3	20.30, 6.644	205.76, 60.985
	(*n* = 351)		
	3 < Aged < 6	21.10, 6.141	197.02, 59.898
	(*n* = 464)		
	Aged > 6	21.80, 5.948	211.19, 61.221
	(*n* = 204)		
	One-way ANOVA	3.900 *	3.891 *
	*F* value		

Note. Separation age means the age of the children at separation from parents. Data are expressed as means (M) and standard deviations (SD). LBC and NLBC represent left-behind children and non-left-behind children, respectively. * *p* < 0.05.

## References

[1] Yuan P., Wang L. (2016). Migrant workers: China boom leaves children behind. Nature.

[2] Lei L.L., Liu F., Hill E. (2018). Labor migration and health of left-behind children in China. J. Dev. Stud..

[3] Zhao C.Y., Zhou X.D., Wang F., Jiang M.M., Hesketh T. (2017). Care for left-behind children in rural China: A realist evaluation of a community-based intervention. Child. Youth Serv. Rev..

[4] Givaudan M., Barriga M., Kercheval J., Pick S. (2016). Children left behind by migration: Training their caretakers. Int. J. Migr. Health Soc. Care.

[5] Wang J., Liu K., Zheng J., Liu J.L., You L.M. (2017). Prevalence of mental health problems and associated risk factors among rural-to-urban migrant children in Guangzhou, China. Int. J. Environ. Res. Public Health.

[6] Nobles J. (2013). Migration and father absence: Shifting family structure in Mexico. Demography.

[7] Zhao X., Chen J., Chen M.C., Lv X.L., Jiang Y.H., Sun Y.H. (2014). Left-behind children in rural China experience higher levels of anxiety and poorer living conditions. Acta Paediatr..

[8] Zhang N., Bécares L. (2016). Chandola, T. A multilevel analysis of the relationship between parental migration and left-behind children’s macronutrient intakes in rural China. Public Health Nutr..

[9] Li B., Chu S., Zhong H.F. (2017). A pilot study on the psychosocial health and living quality of Left-Behind Children in a remote City of China. Health Equity.

[10] Hu H.W., Gao J.M., Jiang H.C., Jiang H.X., Guo S.Y., Chen K., Jin K.L., Qi Y.Y. (2018). A comparative study of behavior problems among left-behind children, migrant children and local children. Int. J. Environ. Res. Public Health.

[11] Barger S.D., Donoho C.J., Wayment H.A. (2009). The relative contributions of race/ethnicity, socioeconomic status, health, and social relationships to life satisfaction in the United States. Qual. Life Res..

[12] Pavot W., Diener E. (2008). The satisfaction with life scale and the emerging construct of life satisfaction. J. Posit. Psychol..

[13] Erdogan B., Bauer T.N., Truxillo D.M., Mansfield L.R. (2012). Whistle while you work: A review of the life satisfaction literature. J. Manag..

[14] Antaramian S. (2015). Assessing psychological symptoms and well-being: Application of a dual-factor mental health model to understand college student performance. J. Psychoeduc. Assess..

[15] Wang S.X. (2014). The effect of parental migration on the educational attainment of their left-behind children in rural China. BE J. Econ. Anal. Policy.

[16] Lu S., Lin Y.T., Vikse J.H., Huang C.C. (2016). Life satisfaction of migrant and left-behind children in China: Education, health, parenting, and personal values. Int. J. Soc. Welf..

[17] Luo Y., Wang H., Lei X., Guo X., Huang K., Liu Q. (2016). Resilience in rural left-behind middle school students in Yunyang County of the Three Gorges area in China: A prospective cohort study. BMC Psychiatry.

[18] Van Luot N., Ba Dat N. (2017). The psychological life satisfaction among Left-Behind Children of labor migrant parents in rural northern Vietnam. Open J. Soc. Sci..

[19] Li L.L., Wang L., Nie J.C. (2017). Effect of parental migration on the academic achievement of Left-behind Middle school students in rural China. China World Econ..

[20] Suldo S.M., Riley K.N., Shaffer E.J. (2006). Academic correlates of children and adolescents’ life satisfaction. Sch. Psychol. Int..

[21] Pavot W., Diener E. (1993). The affective and cognitive context of self-reported measures of subjective well-being. Soc. Indic. Res..

[22] Cai H.J., Huang X.F., Song H.R. (2008). The Relationship between Sex-role and Subjective Life satisfaction in China. Acta Psychol. Sin..

[23] Yang T.T., Li C.C., Zhou C.C., Jiang S., Chu J., Medina A., Rozelle S. (2016). Parental migration and smoking behavior of left-behind children: Evidence from a survey in rural Anhui, China. Int. J. Equity Health.

[24] Huebner E.S., Suldo S.M., Smith L.C., McKnight C.G. (2004). Life satisfaction in children and youth: Empirical foundations and implications for school psychologists. Psychol. Sch..

[25] Cavallo F., Dalmasso P., Ottova-Jordan V., Brooks F., Mazur J., Valimaa R., Gobina I., Gaspar de Matos M., Raven-Sieberer U. (2015). Trends in life satisfaction in European and North-American adolescents from 2002 to 2010 in over 30 countries. Eur. J. Public Health.

[26] Jung S.Y., Choi E. (2017). Life satisfaction and delinquent behaviors among Korean adolescents. Personal. Individ. Differ..

[27] Attiyah A.A.L., Nasser R. (2016). Gender and age differences in life satisfaction within a sex-segregated society: Sampling youth in Qatar. Int. J. Adolesc. Youth.

[28] Hatami G., Motamed N. (2014). Life Satisfaction in children and adolescents with beta thalassemia major in southwest Iran. Electron. Physician.

[29] Katharina R., Max G.H., Klaus H., Matthias R. (2018). Perceived class climate and school-aged children’s life satisfaction: The role of the learning environment in classrooms. PLoS ONE.

[30] Cortina J. (2014). Beyond the money: The impact of international migration on children’s life satisfaction: Evidence from Ecuador and Albania. J. Migr. Dev..

[31] Ye J.Z. (2011). Left-behind children: The social price of China’s economic boom. J. Peasant Stud..

[32] Zhao J.X., Sun P., Wang M.F., Zhang W.X. (2018). Left-behind adolescents’ hopes and fears for the future in rural China. J. Adolesc..

[33] Chang L., Lu H.J. (2018). Resource and extrinsic risk in defining fast life histories of rural Chinese left-behind children. Evol. Hum. Behav..

[34] Hua H.W., Zhu X.R., Jiang H.X., Li Y.Y., Jiang H.C., Zheng P.P., Zhang C., Shang J. (2018). The association and mediating mechanism between poverty and poly-victimization of left-behind children in rural China. Child. Youth Serv. Rev..

[35] Wu Q.B., Cebotari V. (2018). Experiences of migration, parent-child interaction, and the life satisfaction of children in Ghana and China. Popul. Space Place.

[36] Arguillas M.J.B., Williams L. (2010). The impact of parents’ overseas employment on educational outcomes of Filipino children. Int. Migr. Rev..

[37] Bai Y., Zhang L.X., Liu C.F., Shi Y.J., Mo D., Rozelle S. (2018). Effect of parental migration on the academic achievement of left behind children in north western China. J. Dev. Stud..

[38] Lu Y. (2014). Parental migration and education of left-behind children: A comparison of two settings. J. Marriage Fam..

[39] Lv B., Zhou H., Guo X.L., Liu C.H., Liu Z.M., Luo L. (2016). The Relationship between academic achievement and the emotional well-being of elementary school children in China: The moderating role of parent-school communication. Front. Psychol..

[40] Li Q., Liu G.D., Zang W.B. (2015). The health of left-behind children in rural China. China Econ. Rev..

[41] Antaramian S., Lee J. (2017). The importance of very high life satisfaction for students’ academic success. Cogent Educ..

[42] Crede J., Wirthwein L., McElvany N., Steinmayr R. (2015). Adolescents’ academic achievement and life satisfaction: The role of parents’ education. Front. Psychol..

[43] Bücker S., Nuraydin S., Simonsmeier B.A., Schneider M., Luhmanna M. (2018). Subjective well-being and academic achievement: A meta-analysis. J. Res. Personal..

[44] Dimitrova R., Johnson D.J., van de Vijver F.J.R. (2018). Ethnic socialization, ethnic identity, life satisfaction and school achievement of Roma ethnic minority youth. J. Adolesc..

[45] Saha N., Karpinski A.C. (2018). The Influence of social media on international students’ global life satisfaction and academic performance. Student Engagement and Participation: Concepts, Methodologies, Tools, and Applications.

[46] Xu W., Yan N., Chen G., Zhang X., Feng T. (2018). Parent-child separation: The relationship between separation and psychological adjustment among Chinese rural children. Qual. Life Res..

